# Methionine Deficiency Affects Liver and Kidney Health, Oxidative Stress, and Ileum Mucosal Immunity in Broilers

**DOI:** 10.3389/fvets.2021.722567

**Published:** 2021-09-22

**Authors:** Baolin Song, Min Fu, Fang He, Huan Zhao, Yu Wang, Qihang Nie, Bangyuan Wu

**Affiliations:** ^1^College of Life Sciences, China West Normal University, Nanchong, China; ^2^Department of Infectious Diseases and Public Health, Jockey Club College of Veterinary Medicine and Life Sciences, City University of Hong Kong, Hong Kong SAR, China; ^3^Key Laboratory of Southwest China Wildlife Resources Conservation, Ministry of Education P. R. China, Nanchong, China

**Keywords:** methionine deficiency, broilers, liver and kidney injury, antioxidant function, ileum mucosal immunity

## Abstract

Methionine (Met) is the first limiting amino acid in broiler diets, but its unclear physiological effects hamper its effective use in the poultry production industry. This study assessed the effect of a Met-deficient (MD) diet on chicken liver and kidney health, exploring the associated mechanisms of antioxidant capacity and ileum mucosal immunity. Seventy-two broilers were administered either the control diet (0.46% Met in starter diet, 0.36% Met in grower diet) or the MD diet (0.22% Met in starter diet, 0.24% Met in grower diet). Liver and kidney samples were collected every 14 days for anatomical, histological, and ultrastructural analyses, accompanied by oxidative stress assessment. Meanwhile, T- and B-lymphocyte abundance and essential cytokine gene expression were measured in the ileum, the center of the gut**–**liver**–**kidney axis. Signs of kidney and liver injury were observed morphologically in the MD group at 42 days of age. Furthermore, aspartate aminotransferase, alanine aminotransferase, creatinine, and uric acid levels were decreased in the MD group compared with the control group, accompanied by decreased superoxide dismutase activity, increased malondialdehyde content, decreased numbers of T and B lymphocytes, and decreased cytokine expression in the ileum, such as IL-2, IL-6, LITAF, and IFN-γ. These results suggest that MD can induce kidney and liver injury, and the injury pathway might be related to oxidative stress and intestinal immunosuppression.

## Introduction

As the first limiting amino acid in broiler diets and a potential mediator of the immune response, methionine (Met) supplementation is required in poultry production (1). Consumption of a Met-deficient (MD) diet has shown to decrease the body weight, growth speed (2), and abdominal fat (3) in broilers, while appropriate Met supplementation can promote egg production (4) and peripheral blood immunity (5). The chicken body reacts sensitively to Met, which affects feather growth at different ages and causes metabolic changes (6). Many studies have explored more effective use of Met in poultry diets, not just to meet the nutritional needs of the chicken but also as a health product. Assessing the physiological functions of Met through diets supplemented with various MD levels has revealed several negative phenomena. Fouad and El-Senousey (7) reported that an MD diet increased the percentage of abdominal fat due to increased fatty acid synthase and hormone-sensitive lipase activities, lipid accumulation in the liver, and decreased hepatic lipid catabolism (8). The reason why would such fatty acid-related metabolic disorder happen is still unclear. However, since we understand that Met residue is an endogenous substrate in antioxidant defense system and oxidative stress is closely related to fatty acid metabolic gene changes (9), it is possible that Met mainly induces such liver abnormalities in this pathological way. Specifically, oxidative stress is often marked by decreased activities of antioxidant enzymes such as aspartate aminotransferase (AST), alanine aminotransferase (ALT), alkaline phosphatase (ALP), and glutamyl transferase (GGT), which allow downstream oxidation products to attack tissue cells, the first targets of which are mainly parenchymal cells in the liver and renal cells in the kidney (10). Within the cell, the initial stress phase is thought to induce fatty acid metabolism gene changes that result in the structural collapse of mitochondria, microsomes, and peroxisomes (11). Therefore, oxidative stress-related pathology analysis in organelle, cell, and organ levels under MD is urgently needed and might reveal a new injury mechanism that would be helpful for understanding Met and its use as a feed supplement.

Recent studies have reported that the gut–liver–kidney axis plays a vital role in many metabolic diseases, including non-alcoholic fatty liver and steatohepatitis (12, 13). However, the liver and kidney are not the only pathological recipients. Digested substrates in the gut are also able to change the consistency of the intestinal content and induce imbalance in the gut microbiota, which leads to abnormal food digestion. Mucosal immunity, which plays a protective role against pathogenic bacteria, would be impaired, allowing entry of microbial endotoxins into the bloodstream and finally to the liver, where bile is produced and influenced. Following bile secretion into the gut, food and microbes would be influenced for a second time. At the core of this loop, a balanced microbial composition can protect a healthy host from metabolic disorders (14, 15). This process is achieved *via* the bloodstream and is guarded by intestinal mucosal immunity. So, we cannot exclude mucosal immunity as a potential factor affecting the Met pathological process and it would also bring us new ideas about this nutrient. Although increased intestinal cell apoptosis has been reported for broilers consuming MD diets (16), suggesting a possible mucosal immunosuppression, we still understand few about the exact immunity status, especially in the ileum, the cross point in such gut–liver–kidney axis.

In the current study, basal diets were formulated according to a study by Wu et al. (17), in which consumption of an MD diet induced significant immunosuppression in the intestine, and a study by Peng et al. (8), in which significant hepatic fat accumulation was observed using 0.24–0.26% Met in the starter diet and 0.26–0.28% Met in the grower diet. The control Met level was set according to National Research Council (NRC) standards and Mirzaaghatabar et al. (18), who suggested that 0.42–0.45% Met in the starter diet and 0.33–0.40% Met in the grower diet were safe levels. The aim of the study was to assess the kidney and liver health of broilers under MD at the organ, cell, and organelle level, in order to elucidate the pathway of oxidative stress-induced injury. Furthermore, the study aimed to investigate ileum mucosal immunity in broilers under MD, including T-lymphocyte subsets, abundance of B lymphocytes, and essential cytokine expression. The study findings provide new insight into pathological knowledge of MD in broilers.

## Materials and Methods

### Animal Use and Experimental Design

All animal experiments were approved by the Animal Welfare Committee of China West Normal University in accordance with the Laboratory Animal Guidelines for Ethical Review of Animal Welfare (China). This study used a total of 72 one-day old broiler chicks (43 ± 2 g, male) obtained from Cobb Germany (Wiedemar, Germany). The broilers were randomly divided into two treatment groups (control and MD) with six replicates per treatment group and six broilers in each replicate. Six broilers in each replicate were housed in experimental cages (12 cages in total, 2 × 1 × 0.8 m^3^ length/width/height) with free access to feed and water. The room temperature was 28°C ± 2°C (day) and 19°C ± 2°C (night). The experimental period lasted 42 days. Peripheral blood, liver, kidney tissue, and ileum samples were collected on days 14, 28, and 42.

This study first designed starter and grower basal diets composed of ground yellow corn and soybean meal plus supplemental energy, vitamins, and mineral elements, which meet the NRC nutritional standards for broilers, except Met. After calculating the Met content, the basal diets were then used for the MD group, while additional 0.24% and 0.12% DL-Met were added to the starter and grower diets for the control group, respectively. The component details and calculated nutrient levels in these diets are shown in Table 1.

**Table 1:** Diet components of the control and Met deficiency group (%).

**Ingredient**	**Met deficiency diet**		**Control diet**	
	**Starter diet**	**Grower diet**	**Starter diet**	**Grower diet**
	**1 to 21 days**	**22 to 42 days**	**1 to 21 days**	**22 to 42 days**
Ground yellow corn	56.0	59.9	56.0	59.9
Soybean meal	26.6	22.2	26.6	22.2
Soybean oil	2.76	3.60	2.76	3.60
Maize gluten	7.09	7.71	7.09	7.71
Fish meal	3.00		3.00	
Monocalcium phosphate	1.43	1.47	1.43	1.47
Limestone	1.45	1.33	1.45	1.33
Mineral and vitamin premix ^#^	1.00	1.00	1.00	1.00
Salt	0.12	0.24	0.12	0.24
Choline chloride	0.10	0.12	0.10	0.12
L-Lysine (54.6%)	0.10	0.19	0.10	0.19
L-Threonine (98.5%)	0.05	0.06	0.05	0.06
L-Valine (98.0%)	0.03	0.03	0.03	0.03
DL-methionine	0.00	0.00	0.24	0.12
Bentonite	0.24	0.12	0.0	0.0
Nutrient level (%)				
ME, MJ/kg	12.39	12.79	12.39	12.79
Crude Protein	21.17	19.72	21.17	19.72
Lysine	1.19	1.08	1.19	1.08
Methionine	0.22	0.24	0.46	0.36
Met+Cys	0.46	0.49	0.70	0.61
Ca	0.85	0.77	0.85	0.77
Non-phytate P	0.44	0.40	0.44	0.40

# *The premix was made with the following ingredients: iodine, 156 mg; selenium, 25 mg; biotin, 25 mg; folic acid, 167 mg; thiamine, 333 mg; riboflavin, 800 mg; pyridoxine, 417 mg; cobalamin, 2.5 mg; nicotinamide, 6.91 g; calcium, 300 g; chloride, 1.0 g; retinol, 1,200,000 IU; cholecalciferol, 400,000 IU; menadione, 333 mg; calcium pantothenate, 2.0 g; choline chloride, 40 g; iron, 5.0 g; copper, 1.5 g; manganese, 10.0 g; and zinc, 7.0 g. All values presented above were calculated using AMINODat ® 5.0*.

### Morphological Observation

#### Body Weight

A total of 12 broilers were randomly chosen at 7, 14, 21, 28, 35, and 42 days for body weight to avoid the sample number change by the weekly chicken euthanasia. The body weight data were gained using an electronic scale, and the weekly weight gain were calculated shown in Table 2.

**Table 2:** Body weight changes of broilers in the control and methionine deficiency group.

**Experimental time**	**Body weight gain in the control group (g)**	**Body weight gain in the methionine-deficient group (g)**	**SEM**	***p*-value**	**Sample number**
7 days	135.22	128.85	0.758	0.755	72
14 days	295.77	277.42	3.227	0.158	60
21 days	415.35	370.28^*^	9.554	0.032	60
28 days	552.43	507.42^**^	17.251	0.009	48
35 days	625.72	535.64^**^	26.758	0.001	48
42 days	705.55	601.42^**^	29.105	0.000	36

Body weight gain data are shown as the mean in the table. The sample numbers were 12 at all the experimental times mentioned above. Significant differences are labeled with * or **,

* *p < 0.05, compared with the control group*,

** *p < 0.01, compared with the control group*.

#### Anatomical, Histological, and Ultrastructural Observations

At 14, 28, and 42 days of age, 12 broilers in each group were randomly chosen and euthanized before being photographed using a color video camera (3CCD; Nikon, Shanghai, China). After anatomical observation, the kidneys and liver were excised and fixed in 4% paraformaldehyde solution for histological observation. After rinsing with water, the samples were dehydrated in a graded series of absolute ethanol (50, 70, 80, 90, and 100%), cleared with benzene twice, followed by saturation and embedding in paraffin. Sections 5 μm thick (10 slices per sample) were prepared and stained with hematoxylin and eosin. A 4XC-W light microscope (Olympus, Tokyo, Japan) was used to observe histological changes in the liver and kidney, and images were edited using Image-Pro software (Media Cybernetics, Rockville, MD, USA). At 42 days of age, kidney and liver samples were collected for ultrastructural observation. The samples were fixed in 2.5% glutaraldehyde, post-fixed in 2% veronal acetate-buffered OsO_4_, dehydrated with alcohol, and embedded in Araldite resin. Sections 65–75 nm thick were placed in uncoated copper grids and stained with uranyl acetate and 0.2% lead citrate. Images were collected using an H-600 transmission electron microscope (Hitachi, Tokyo, Japan) and edited using Image-Pro software.

#### Metabolic Function Assessment Through Peripheral Blood

Peripheral blood was collected from chickens every 14 days, from which the serum was obtained. Related enzyme activities, such as aspartate transaminase (AST), alanine transaminase (ALT), alkaline phosphatase (ALP), and gamma-glutamyltransferase (GGT), were determined by ultraviolet spectrophotometry, using a microplate reader and colorimetric methods. Serum was also used to assess creatinine (Cr), uric acid (UA), and blood urea nitrogen (BUN) levels using the sarcosine oxidase method, enzyme colorimetry, and urease method, respectively. All methods were performed using reagent kits purchased from Nanjing Jiancheng Bioengineering Institute (Nanjing, China).

#### Determination of Antioxidant Ability of Kidney and Liver

Every 14 days, fragments of collected kidney and liver samples were homogenized and centrifuged at 3,500 rpm for 10 min. The supernatant was collected and superoxide dismutase (SOD), catalase (CAT), and glutathione peroxidase (GSH-Px) activities were determined using the hydroxylamine method (for SOD) and colorimetric methods (CAT and GSH-Px). Additionally, glutathione (GSH) content was measured using a spectrophotometric method and malondialdehyde (MDA) content was determined using the thiobarbituric acid (TBA) method. All methods were performed using reagent kits purchased from Nanjing Jiancheng Bioengineering Institute.

### Assessment of Intestine Mucosal Immunity

#### Detection of T-Lymphocyte Subsets in Ileum

Isolation of intraepithelial lymphocytes (IELs) from the ileum was carried out as described by Montufar-Solis and Klein (19) and Todd et al. (20). Briefly, ileum samples were dissected, washed with D-Hank's buffer, cleaned with phosphate-buffered saline (PBS) without Ca^2+^ and Mg^2+^, and digested using a solution consisting of ethylenediaminetetraacetic acid (EDTA), dithiothreitol (DDT), and PBS. After incubation at 37°C followed by centrifugation, the cells were collected and analyzed by flow cytometry.

Resendiz-Albor's isolation method was used to obtain lamina propria lymphocytes (LPLs) from the ileum (21). Briefly, after IELs were collected, the samples were treated with EDTA, cleaned, and transferred to centrifuge tubes containing collagenase, fetal bovine serum (FBS), and gentamicin. After shaking and centrifugation, the suspension containing the lamina propria cells was filtered and centrifuged again, after which the LPLs were collected and prepared for flow cytometry.

The collected IELs and LPLs were made into a cell suspension and transferred into the flow tube. Mouse anti-chicken CD4-FITC (MCA2164F), CD3-SPRD (8200-13), and CD8-RPE (GTX74921) antibodies were added in turn, and stained in the dark at 4°C for 30 min. After washing with PBS, 500 μl of PBS was added to the flow tube and cells were detected by using a BD FACS flow cytometer (Becton, Dickinson and Company, Franklin Lakes, NJ, USA) for 1 h. Lymphocyte subset data were calculated as the number of target positive cells in the whole group of T cells by percentage.

#### Detection of B Cells by Immunohistochemistry (IHC)

Paraffin sections of ileum samples were deparaffinized, blocked for endogenous peroxidase by incubating with 3% H_2_O_2_, boiled with 0.01 M sodium citrate buffer for 20 min, and cooled at room temperature. The slices were subsequently incubated in 5% bovine serum albumin (BSA), and then incubated with mouse anti-chicken immunoglobulin (Ig) A antibody followed by incubation with biotinylated secondary antibody goat anti-mouse Ig (GNBP2-34648B, Novus Biologicals, Little Rock, CO, USA). Finally, the slices were incubated with streptavidin-biotin complex at 37°C. After washing, the slices were stained with DAB-H_2_O_2_, counterstained with hematoxylin, dehydrated in ethanol, and sealed with neutral gum. The number and distribution of IgA-positive cells in the ileum were observed under a light microscope. Five slices were made for each intestinal segment of each broiler, and the same parts of the ileum were analyzed in each slice. Images were collected and edited using Image-Pro software.

#### Detection of CD19 by ELISA

Ileum segments were treated using the same method as described above. CD19 content was detected in the sample homogenate using the CD19 ELISA kit (Jianglai Biological Co., Ltd., Shanghai, China), according to the manufacturer's instructions. The absorbance (OD) of the treated sample was measured at 450 nm and the CD19 level was calculated using a standard curve.

#### Detection of Cytokine Expression by qRT-PCR

Ileum segments stored at −195.8°C were ground with liquid nitrogen until a uniform powder was obtained. Total RNA was isolated using RNAiso Plus (Takara Bio, Kumatsu, Japan) and reverse transcribed to cDNA using the Prim-Script™ RT Reagent Kit (Takara Bio). cDNA was used as the PCR template. Oligonucleotide primers were designed using Primer 5 software and synthesized by Takara Bio (Dalian, China), as shown in Table 1. The qRT-PCR reactions (25 μl total volume) included 12.5 μl of SYBR® Premix Ex Taq™ II (Takara Bio, Japan), 1 μl of forward and 1 μl of reverse primers, 8.5 μl of RNase-free water (Tiangen Biotech, Co., Ltd., Beijing, China), and 1 μl of cDNA. A C1000 Touch Thermal Cycler (Bio-Rad, Hercules, CA, USA) was used to perform qRT-PCR. The thermal cycling conditions were as follows: 95°C for 3 min, followed by 44 cycles of 95°C for 10 s, Tm of the specific primer pair for 30 s, and 95°C for 10 s, followed by 72°C for 10 s. Melting curve analysis displayed only one peak for each PCR product. Chicken β-actin was used as the internal reference housekeeping gene. Gene expression values from the control group samples at 14, 28, and 42 days were used to calibrate gene expression values for MD group samples. Expression fold changes were calculated using the 2^−ΔΔCT^ method, as described by Ochshorn et al. (22). The details of cytokine mRNA expression detection using primers and normalization primer sequences are provided in Supplementary Table 1.

### Statistical Analysis

Statistical analysis was performed using Microsoft Excel and IBM SPSS Statistics v19 software (IBM Corp., Armonk, NY, USA). Differences between treatment groups were analyzed by independent sample *t*-test, and the results are presented as mean ± standard deviation (SD). Differences between means were assessed, and values of *p* < 0.05 were considered significant, while values of *p* < 0.01 were considered extremely significant between the control and MD groups.

## Results

### Growth Performance of Broilers Was Negatively Affected by MD

Although the body weight gains of broilers in both control and MD groups increased continuously for 42 days, the upward trend of data in MD groups is suppressed significantly showing a body growth inhibition. The mean weight gain data in the control group begin to be significantly higher than them in the MD group from 14 days of age (*p* < 0.05), and the difference finally reaches a very significant level from 21 days of age (*p* < 0.01).

### Mitochondrial Damage, Cell Degeneration, and Organ Swelling Induced by MD in Liver and Kidney

**Figure 1:**
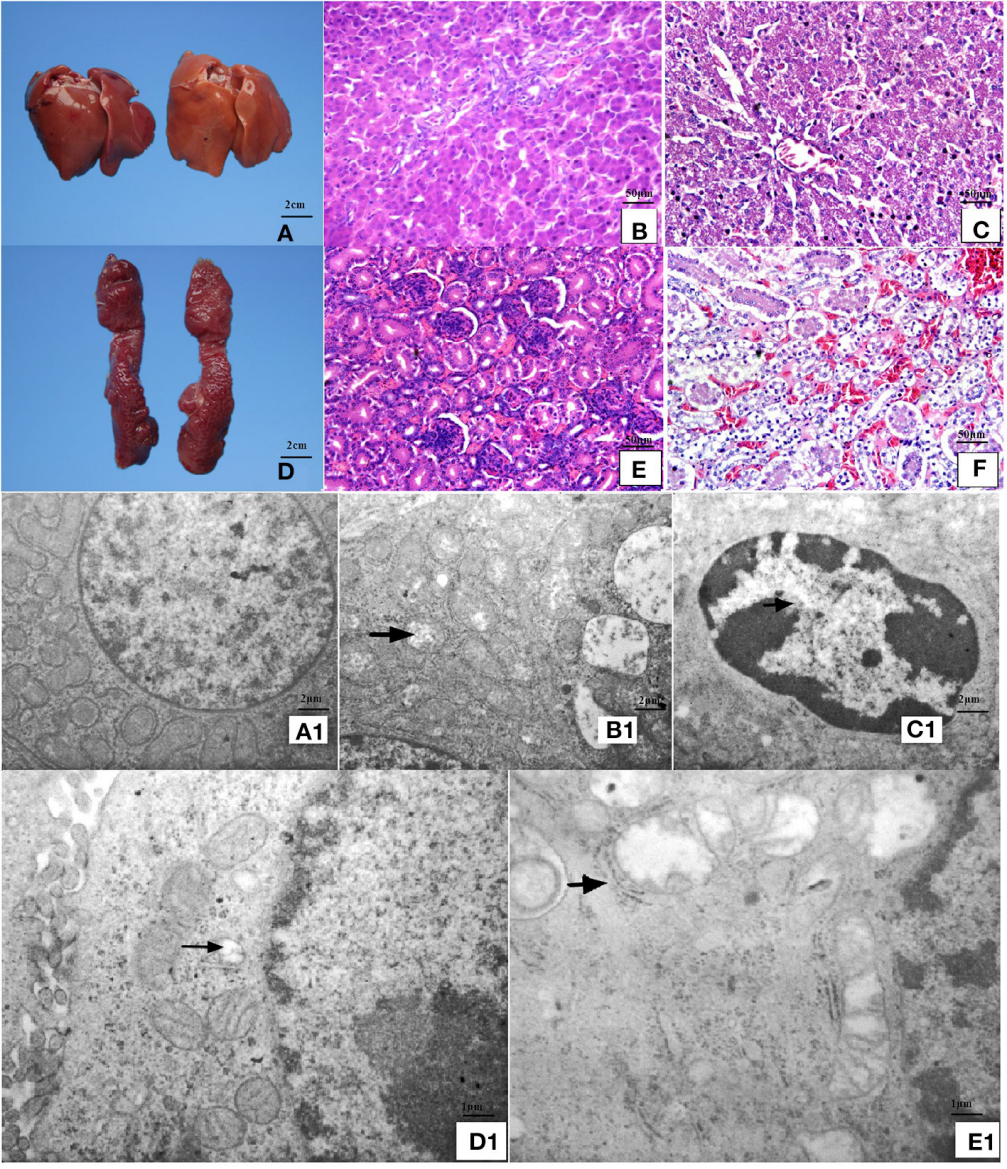
Anatomical, histopathological, and ultrastructural observations of liver and kidney. **(A)** The liver was swollen, blunt-edged, and yellow and **(D)** the kidney was swollen in the MD group (right side of panels) at 42 days of age compared to the control group (left side of panels). **(C)** Severe granular degeneration in liver cells was observed in the MD group at 42 days of age (H&E staining ×400) compared to **(B)** the control group (H&E staining ×400). **(F)** Vacuolar degeneration was observed in kidney cells with lots of liquid entry into the cytoplasm in the MD group at 42 days of age (H&E staining ×400) compared to **(E)** the control group (H&E staining ×400). **(A1)** Hepatocytes in the control group possessed clear bilayer lipid membranes and normal organelles (TEM×12,000). **(B1)** The mitochondria of hepatocytes in the MD group were swollen or vacuolated with degenerating cristae (left arrow) (TEM×12,000). **(C1)** The chromatin of hepatocytes in the MD group was condensed (left arrow) (TEM×25,000). **(D1)** Renal cells in the control group possessed normal organelles (TEM×25,000). **(E1)** The mitochondria of hepatocytes in the MD group were swollen or vacuolated with degenerating cristae (left arrow) (TEM×25,000). MD, methionine deficiency; H&E, hematoxylin and eosin; TEM, transmission electron microscopy.

Chicks fed an MD diet for 42 days exhibited liver and kidney injury (Figure 1). Anatomical observations revealed that the liver was swollen, blunt, round-edged, turbid, lusterless, and grayish-yellow, as shown in Figure 1A. These features were speculated to be correlated with granulosis or steatosis, which was supported by the histological and ultrastructural observations. The liver cells were disordered with severe granular degeneration into the cytoplasm (Figure 1C), accompanied by distortion and swelling of the mitochondria (Figures 1B1,C1). The histological observations of the kidney were more meaningful than the anatomical observations. Only slight swelling and blunt edges were observed in the kidney (Figure 1F), but histological examination revealed extreme vacuolar degeneration and hyperemia in renal tubule cells, accompanied by serious degeneration of mitochondria (Figures 1D1,E1). All liver- and kidney-related morphological observations indicated severe injury at the organ, cell, and organelle levels.

### MD Leads to Metabolic Disorder in Growing Chicks

**Figure 2:**
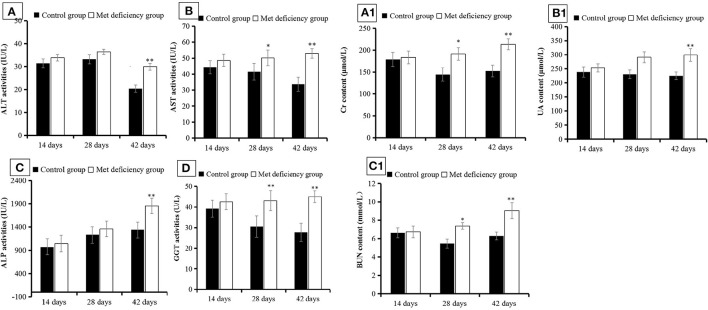
Liver and kidney function detection. The activities of **(A)** ALT, **(B)** AST, **(C)** ALP, and **(D)** GGT were significantly (*p* < 0.05, *p* < 0.01) increased at 42 days of age in the MD group compared to the control group. Levels of **(A1)** Cr, **(B1)** UA, and **(C1)** BUN were all significantly (*p* < 0.05 or *p* < 0.01) increased at 28 or 42 days of age in the MD group compared to the control group. ALT, alanine transaminase; AST, aspartate transaminase; alkaline phosphatase; GCT, gamma-glutamyl transferase; MD, methionine deficiency; Cr, creatine; UA, uric acid; BUN, blood urea nitrogen.

AST (*p* < 0.05) and GGT (*p* < 0.01) activities were significantly higher in the MD group than in the control group at 28 days of age and continued to increase over the experimental period (*p* < 0.01) (Figures 2B,D). ALT and ALP activities did not differ significantly between the treatment groups at 28 days of age (*p* > 0.05), but were significantly higher in the MD group at the end of the experimental period compared to the control group (*p* < 0.01) (Figures 2A,C). This indicated that liver injury was aggravated over time, concentrated mostly between 28 and 42 days of age. In addition, Cr, BUN, and UA serum levels in the MD group also increased significantly compared to the control group, reaching maximum values at 42 days of age (*p* < 0.01) (Figures 2A1–C1). These results indicated severe renal cell injury.

### MD Leads to Oxidative Stress in Liver and Kidney of Growing Chicks

**Figure 3:**
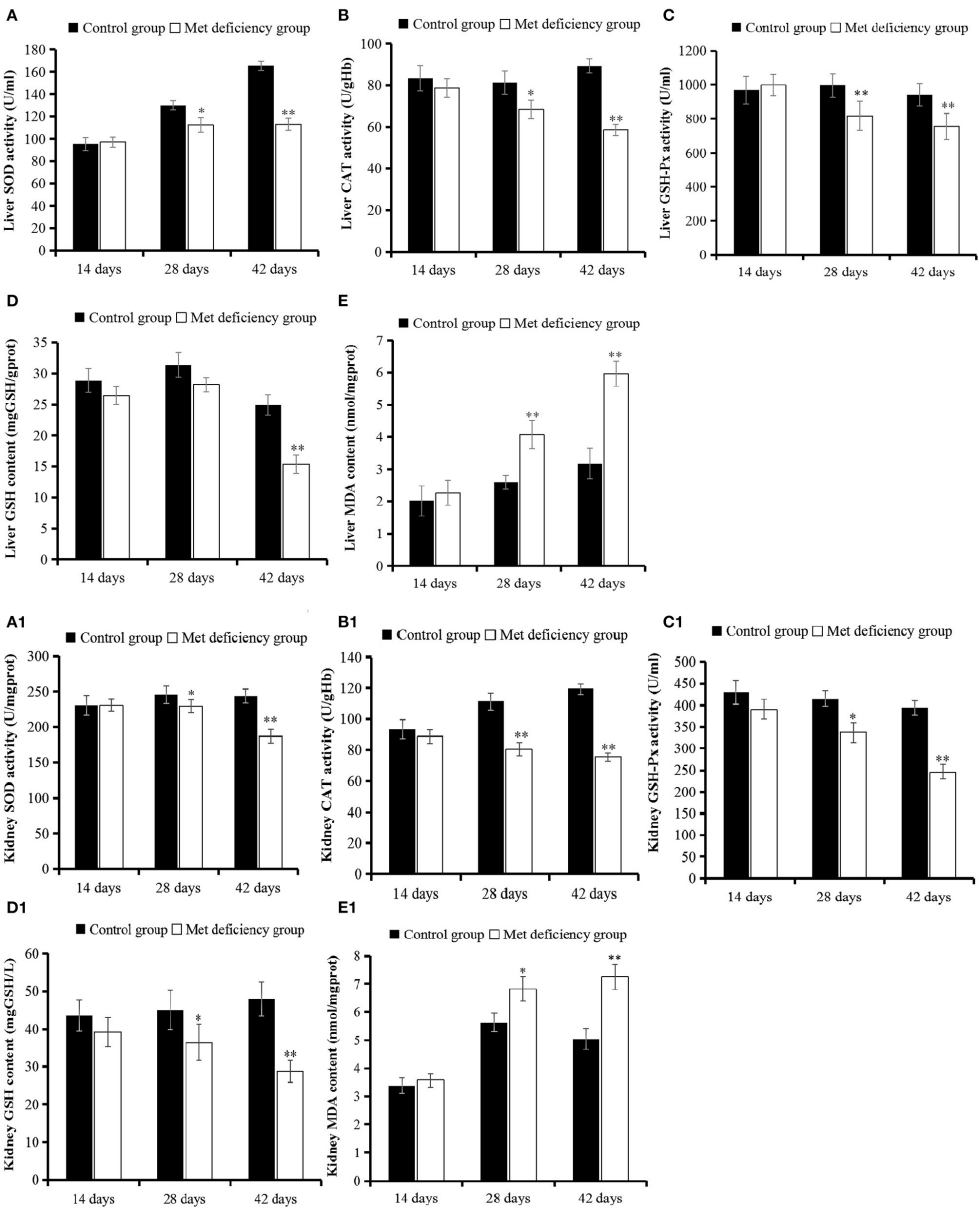
Oxidative stress evaluation in liver and kidney. The activities of SOD, CAT, and GSH-Px in liver **(A–C)** and kidney **(A1–C1)** were all significantly (*p* < 0.05 or *p* < 0.01) decreased at 28 or 42 days of age in the MD group compared to the control group. **(D** and **D1)** The level of antioxidant substrate GSH decreased in the MD group at 42 days of age compared to the control group. **(E** and **E1)** The level of oxidation product MDA increased significantly in the MD group at 28 or 42 days of age. SOD, superoxide dismutase; CAT, catalase; GSH-Px, glutathione peroxidase; MD, methionine deficiency; MDA, malondialdehyde; GSH, glutathione.

As shown in Figure 3, the level of oxidation product MDA significantly increased in both the liver and kidney of the MD group at 42 days of age compared to the control group (*p* < 0.01). However, the activities of antioxidant enzymes SOD, GHS-Px, and CAT and the level of antioxidant GSH were significantly decreased at 42 days of age (*p* < 0.01) in the MD group compared to the control group.

### MD Inhibits Ileum Intraepithelial and Lamina Propria T-Cell Immunity Establishment in Growing Chicks

**Figure 4:**
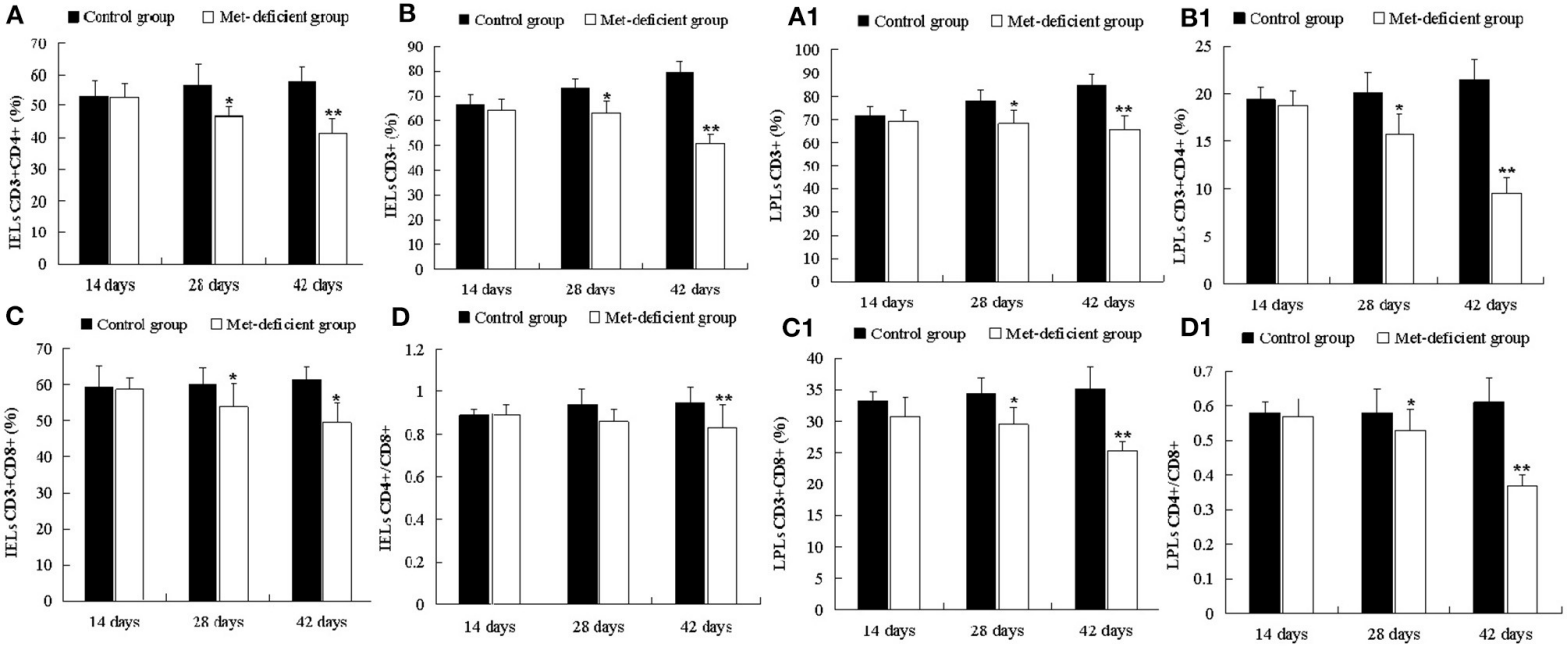
Changes in the abundance of ileac CD3+, CD3+CD4+, CD3+CD8+, and CD4+/CD8+ T IELs and LPLs in broilers. At 28 and 42 days of age, the CD3+ **(B)**, CD3+CD4+ **(A)**, and CD3+CD8+ **(C)** IEL populations in the MD group were significantly decreased (*p* < 0.05 or *p* < 0.01), and the CD4+/CD8+ **(D)** IEL population was significantly decreased (*p* < 0.01) at 42 days of age compared to the control group. At 28 and 42 days of age, the CD3+ **(B1)**, CD3+CD4+ **(A1)**, CD3+CD8+ **(C1)**, and CD4+/CD8+ **(D1)** LPL populations in the MD group were significantly lower than those in the control group (*p* < 0.05 or *p* < 0.01). IELs, intraepithelial lymphocytes; LPLs, lamina propria lymphocytes; MD, methionine deficiency.

As shown in Figures 4A–D, the percentages of IELs CD3+, CD3+CD4+, and CD3+CD8+ in the MD group decreased significantly at 28 (*p* < 0.05) and 42 days of age (*p* < 0.01) compared to the control group. Furthermore, the percentage of IELs CD4+/CD8+ was significantly decreased (*p* < 0.01) at 42 days of age. These results suggested that MD affected the abundance of IEL subsets and reduced the ability of the chick immune system to defend against pathogens. Moreover, LPLs constitute part of the mucosal immune response. The LPL subsets demonstrated similar trends to those of the IEL subsets. LPL CD3+, CD3+CD4+, and CD3+CD8+ percentages were significantly lower in the MD group at 28 days of age than in the control group (*p* < 0.05), while the percentage of LPLs CD4+/CD8+ in the MD group was significantly lower than that in the control group at 42 days of age (*p* < 0.01) (Figures 4A1–D1).

### MD Limits the Activation, Proliferation, and Number of B Cells in the Ileum of Growing Chicks

**Figure 5:**
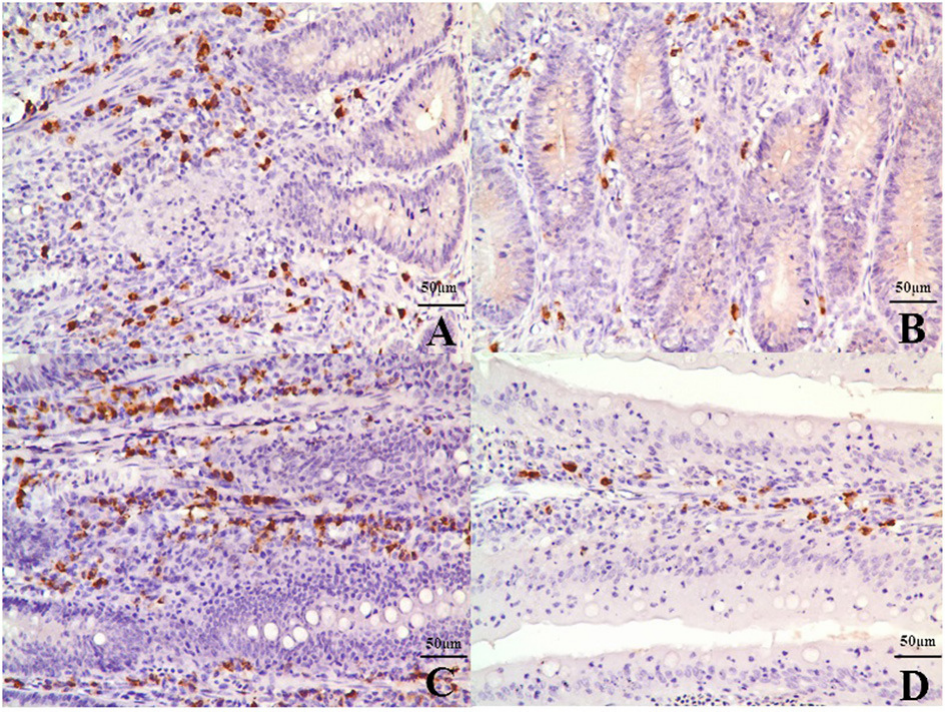
IgA-positive B cells in the submucosa and lamina propria in the villi of the ileum at 42 days of age. **(A)** The ileac submucosa of the control group (400×, bar = 50 μm). **(B)** The quantity of B cells in the ileac submucosa of the MD group was significantly decreased (400×, bar = 50 μm). **(C)** The lamina propria in the villi of the ileum in the control group (400×, bar = 50 μm). **(D)** The quantity of B cells in the lamina propria in the villi of the ileum in the MD was significantly decreased (400×, bar = 50 μm). MD, methionine deficiency.

The IHC results revealed that IgA-positive B cells were mainly distributed in the submucosa and lamina propria in the villi of the ileum (Figures 5A–D) with similar numbers of B cells in both treatment groups at 14 days of age. However, the number of B cells in the MD group decreased significantly at 28 (*p* < 0.05) and 42 days of age (*p* < 0.01) compared to the control (Supplementary Figure 1). Cell inactivation is indicated by mass suppression of cytokine expression. As shown in Supplementary Figure 2, the mRNA expression of IL-2, IL-6, IL-10, IL-17, IFN-γ, and LITAF decreased significantly at 28 (*p* < 0.05) and 42 days of age (*p* < 0.01) in the MD group compared to the control group.

## Discussion

Met supplementation for broilers is important before 42 days of age, when they are growing quickly and establishing immunity. Peng et al. (8) reported that broiler consumption of an MD diet with 0.3% Met increased lipid accumulation in the liver by decreasing hepatic lipid catabolism, which concurred with reported findings of liver weight gain in broilers with a 0.2% Met diet (23) and abnormal increased triglyceride levels and decreased lipoprotein-cholesterol levels in serum of broilers with a 0.3% Met diet (24). For the kidney, the organ most closely related to the liver, renal cell apoptosis in broilers was upregulated through the P53–P21 cancer gene pathway with consumption of a 0.3% Met diet (25). Our study starts with MD diets containing 0.24 and 0.26% Met in the starter and grower diets, respectively. So, the pathological changes revealed in this study would not be aggravated significantly due to Met content differences.

The study demonstrated the effect of MD on the growth performance of broilers. Growth speed under MD was significantly reduced, which was consistent with the conclusion that a 0.2% Met diet would be harmful for chicken growth and meat quality (26). This demonstrates that the broiler chicken metabolism was affected apparently. Through morphological observations, it is found that the kidney and liver were both swollen, blunt-edged, and turbid, and the kidney had turned yellow (Figure 1), suggesting that both organs were injured and significant lipid accumulation might have occurred in the liver, which may also reflect the lipid metabolism disorder found before this study (24). However, the histopathological results indicated granulosis in the liver rather than steatosis (Figure 1), suggesting that lipid accumulation was not as severe as previously thought. In contrast, the granulosis and mitochondrial fold destruction found *via* ultrastructural observations suggested more serious metabolic disorder led by mitochondrial injury. This can also be supported by indicators (27, 28) including ASL, ALT, ALP, and GGP activities in the liver (Figure 2), and Cr, UA, and BUN levels in the kidney (Figure 2). Because only when destructive injury happens such as mitochondrial collapse can these contents be released instead of lipid accumulation inside liver cells. So, the lipid metabolic disorder may be the secondary phenomenon of such pathological changes while mitochondrial injury is induced first. As mentioned by a study (8), lipid metabolism-related gene changes can be influenced in a moderate level by oxidative stress. We need to discuss further whether the mitochondrial injury achieved a significant oxidative stress.

**Figure 6:**
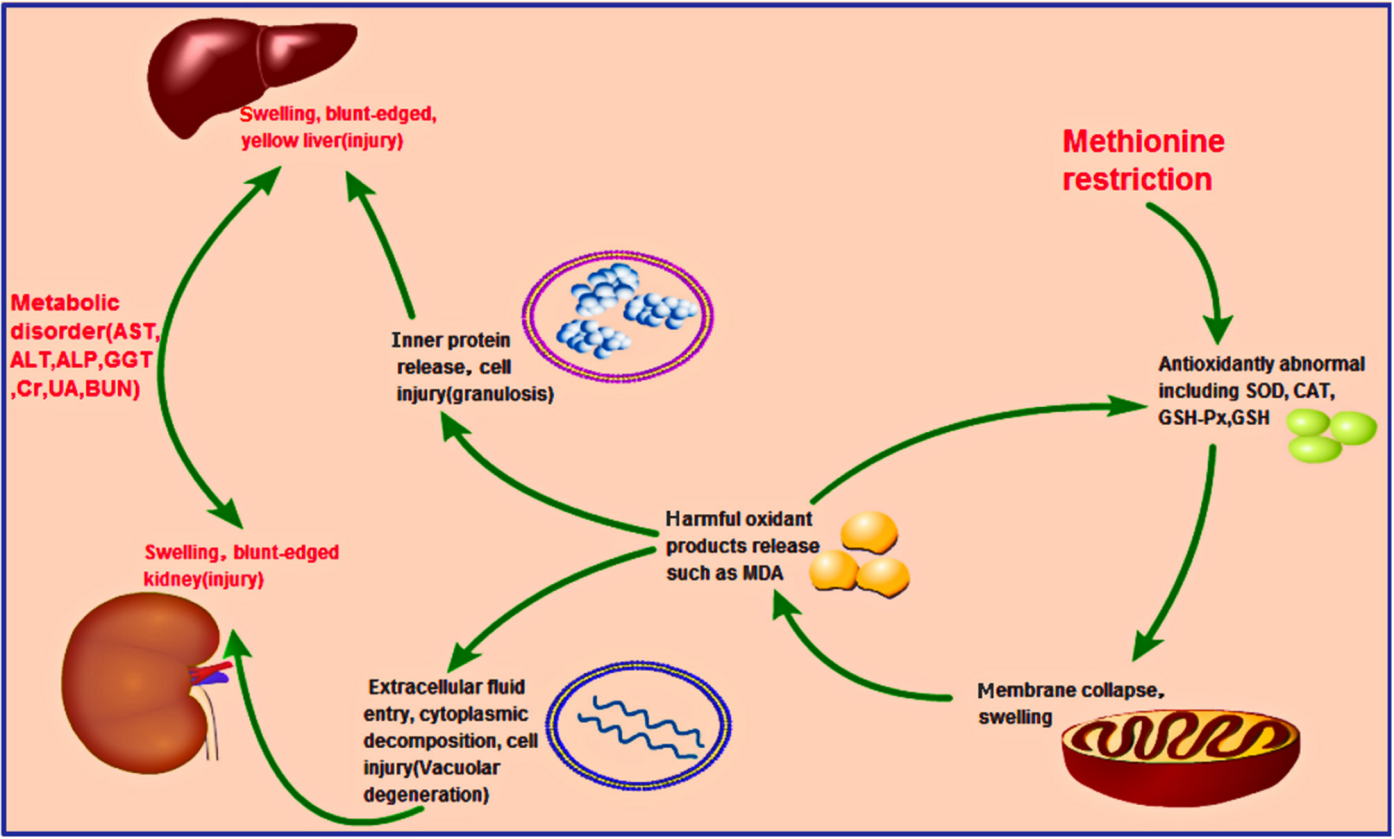
Hypothesized pathology of methionine deficiency in the liver and kidney, reflecting induced oxidation stress. Methionine deficiency can decrease the activities of antioxidant enzymes such as SOD, CAT, GSH-Px, and GSH. The mitochondria, where many oxidation-related chemicals such as MDA exist, would be damaged, resulting in their release into the cytoplasm due to membrane collapse. Other organelles in the liver and kidney would then be harmed, releasing lots of proteins and changing the osmotic pressure, causing granulosis degeneration (liver) and vacuolar degeneration (kidney), respectively. Prolonged methionine deficiency could induce liver and kidney injury, finally leading to metabolic disorder.

The study findings suggested some latent reasons, including the occurrence of oxidative stress. Met is also known as the precursor of succinyl-CoA, homocysteine, cysteine, creatine, and carnitine (29), some of which are essential substrates for antioxidant molecules, such as GSH. These molecules prevent oxidative stress (30), which is extremely harmful to cells and may cause cell death and tissue damage. We analyzed more key enzymes and products in the antioxidant loop to obtain a broader view of oxidative status in broilers. As shown in Figure 3, the GSH content in the liver and kidney decreased significantly (*p* < 0.05) from 14 days of age and complies with the results of a study by Swennen et al. (31). Furthermore, significant lower (*p* < 0.01) antioxidant enzyme activities (SOD, CAT, and GSH-Px) in the liver and kidney were detected in growing chicks in the MD group than in the control group at 42 days of age (Figure 3). Moreover, levels of the oxidation product MDA increased significantly (*p* < 0.01) at 42 days of age in the MD group (Figure 3). This evidence clarifies the existence of oxidative stress in broilers. These findings might explain the results of mitochondrial membrane potential collapse (Figure 1) and metabolic disorder (Figure 2), because structural alterations of proteins and mitochondrial DNA (32) are the initial part of oxidative stress. The discussed possible pathway is shown in Figure 6.

**Figure 7:**
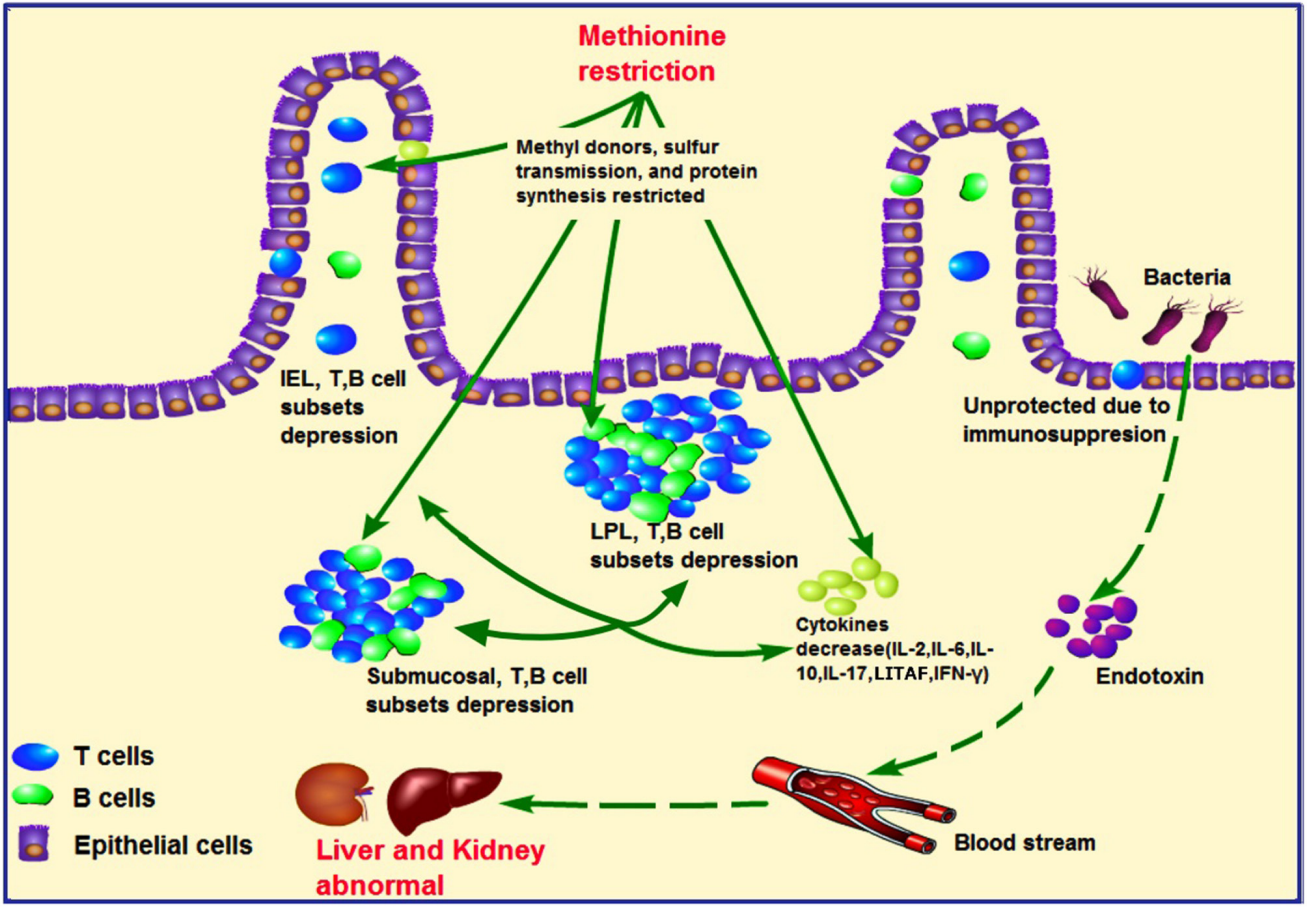
Hypothesized pathology of methionine deficiency in ileum mucosal immunity, and the latent pathway of liver and kidney injury. Methionine, an important methyl donor and source of sulfur, might influence protein synthesis and affect intestinal immunity. Under methionine deficiency, IELs and LPLs and submucosal T- and B-cell subsets were suppressed, which could be partially responsible for decreased levels of cytokines such as IL-2, IL-6, IL-10, LITAF, and IFN-γ. These phenomena contribute to ileum mucosal immunosuppression. As intestinal immunity plays a central role in the gut**–**liver**–**kidney axis, uncontrolled harmful bacteria might lead to abnormal liver and kidney function due to secretion of endotoxins. IELs, intraepithelial lymphocytes; LPLs, lamina propria lymphocytes.

The kidney and liver injury found in the current study might also be correlated with compromised immunity in the ileum. MD has been shown to decrease the immune function of chicks. MD can inhibit the development the bursa of Fabricius (33), while increasing cell apoptosis and cell cycle arrest in the spleen, accompanied by oxidative stress (17). However, studies investigating the immunity of the intestine under these conditions are lacking. In addition, further evidence has shown that crosstalk occurs between intestinal mucosal immunity and gut microbes. When the intestinal immune bridge breaks, kidney metabolism disorders may result (34, 35). In our study, the abundances of IEL and LPL subsets were found to be decreased in the MD group (Figure 5), including CD3+, CD4+, or CD8+ cells (Figure 4), in agreement with reported findings of their T-cell function (36). In addition, IL-2, IL-6, IL-10, IL-17, LITAF, and IFN-γ (Supplementary Figure 2) expression also decreased significantly in the MD group, suggesting impaired activating ability and efficiency of ileum mucosal immunity. This was also supported by the activation, proliferation, and number of B cells in the MD group (Supplementary Figure 1). So, in regard to cytokine content decrease and LEL and LPL lymphocyte depression, the LEL and LPL subset suppression occurs in the MD broilers. The mechanism responsible for such changes might be related to Met's function as a methyl donor and its involvement in sulfur transmission and protein synthesis, which could cause abnormal gene expression and cell death (37). The ileum is where blood from the intestine returns to the liver through the portal vein. Due to severe immunosuppression found in this key location, we suggest that intestinal immunosuppression played a role in the observed liver and kidney abnormalities. Therefore, we hypothesize that MD-induced liver and kidney injury might also be achieved *via* a second pathway by the gut**–**liver**–**kidney axis described also in Figure 7.

In conclusion, MD directly affects the growth performance of broilers reflected by suppressed growth rate. The physiological mechanisms responsible may be related to liver and kidney injury due to metabolic disorder achieved *via* increased oxidative stress in these organs and mucosal immunosuppression in the ileum. Additional studies investigating mucosal immunity or microbes between metabolic organs and the gut are warranted to better evaluate Met diet standards.

## Data Availability Statement

The datasets presented in this study can be found in online repositories. The names of the repository/repositories and accession number(s) can be found in the article/Supplementary Material.

## Ethics Statement

The animal study was reviewed and approved by The Animal Welfare Committee of China West Normal University.

## Author Contributions

BS: Conceptualization, Validation, and Formal Analysis. MF, FH, and HZ: Methodology. YW, QN and HZ: Software. BS and MF: Investigation. YW and QN: Data Curation. BS and BW: Writing—Original Draft Preparation and Visualization. BW and HZ: Writing—Review and Editing. BW: Supervision. All authors contributed to the article and approved the submitted version.

## Funding

This study is supported by the program for the Fundamental Research Funds of China West Normal University (Project No. 20A003), the Meritocracy Research Funds of China West Normal University (Project No. 17YC349), and the Education Department of Sichuan Province (Project No. 17ZB0425).

## Conflict of Interest

The authors declare that the research was conducted in the absence of any commercial or financial relationships that could be construed as a potential conflict of interest.

## Publisher's Note

All claims expressed in this article are solely those of the authors and do not necessarily represent those of their affiliated organizations, or those of the publisher, the editors and the reviewers. Any product that may be evaluated in this article, or claim that may be made by its manufacturer, is not guaranteed or endorsed by the publisher.
