# Supplementary material for: Methionine Deficiency Affects Liver and Kidney Health, Oxidative Stress, and Ileum Mucosal Immunity in Broilers
Source: Front Vet Sci. 2021 Sep 22;8:722567. doi: 10.3389/fvets.2021.722567 (PMC8493001; doi:10.3389/fvets.2021.722567)
Supplement: Supplementary file 1 [file Data_Sheet_1.docx]

Supplementary Material

# Supplementary Tables

**Supplementary Table S1.**

A list of oligonucleotides used as primers in qRT-PCR analysis of MDNA expression in ileum.

| Gene symbol | Accession number | Primer | Primer sequence (5′→3′) |
| --- | --- | --- | --- |
| IL-2 | AF000631 | F | TCTGGGACCACTGTATGCTCT |
|  |  | R | ACACCAGTGGGAAACAGTATCA |
| IL-6 | AJ309540 | F | CAAGGTGACGGAGGAGGAC |
|  |  | R | TGGCGAGGAGGGATTTCT |
| IL-10 | AJ621614 | F | CGGGAGCTGAGGTGAA |
|  |  | R | GTGAAGAAGCGGTGACAGC |
| IL-17 | AJ493595 | F  R | CTCCGATCCCTTATTCTCCTC  AAGCGGTTGTGGTCCTCAT |
| IFN-γ | Y07922 | F | AGCTGACGGTGGACCTATTATT |
|  |  | R | GGCTTTGCGCTGGATTC |
| LITAF (LITAF) | AY765397 | F | TGTGTATGTGCAGCAACCCGTAGT |
|  |  | R | GGCATTGCAATTTGGACAGAAGT |
| β-Actin | L08165 | F | TGCTGTGTTCCCATCTATCG |
|  |  | R | TTGGTGACAATACCGTGTTCA |

All qRT-PCR related data were normalized by β-Actin gene expression results, and the figures are expressed by △△CT.

##
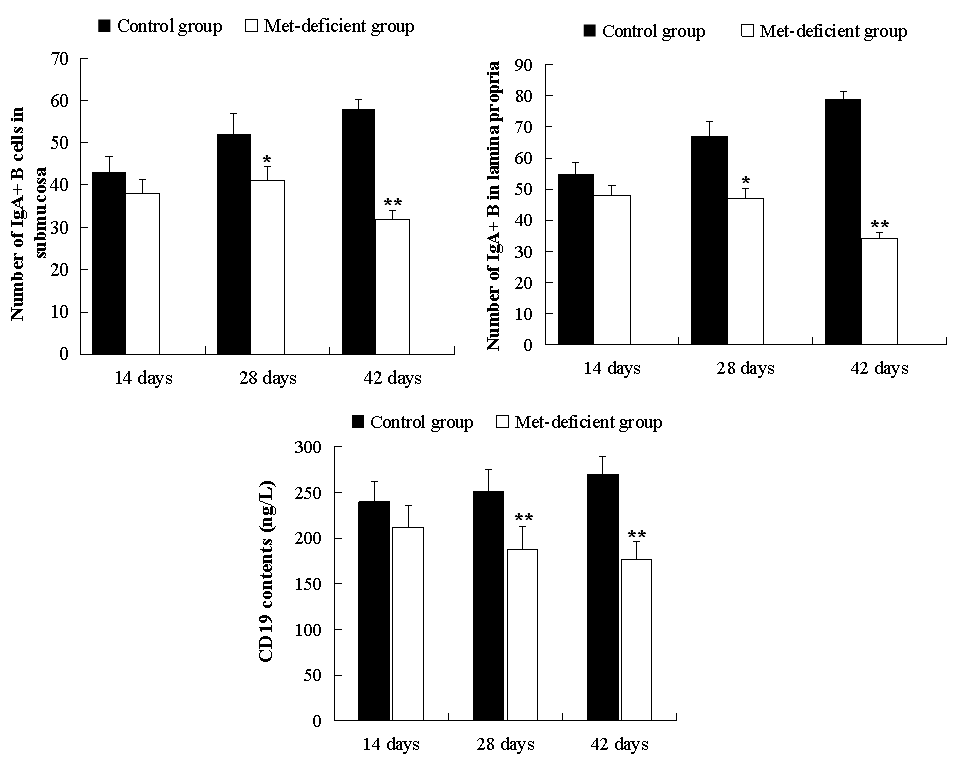
Supplementary Figures

**Supplementary Figure S1. Changes of the CD19 content and the positive B cell numbers in the submucosa and lamina propria in the villi of the ileum (n=6).**The content of CD19 decreased significantly in the ileum at the 28, and 42 days of age (p < 0.01) in the methionine deficiency group, compared to that in the control group. And, the numbers of IgA+ B cells decreased at the same period of time (p< 0.05, p < 0.01), whether in the submucosa and in the lamina of Propria. *p<0.05, compared with the control group; **p<0.01, compared with the control group.

**
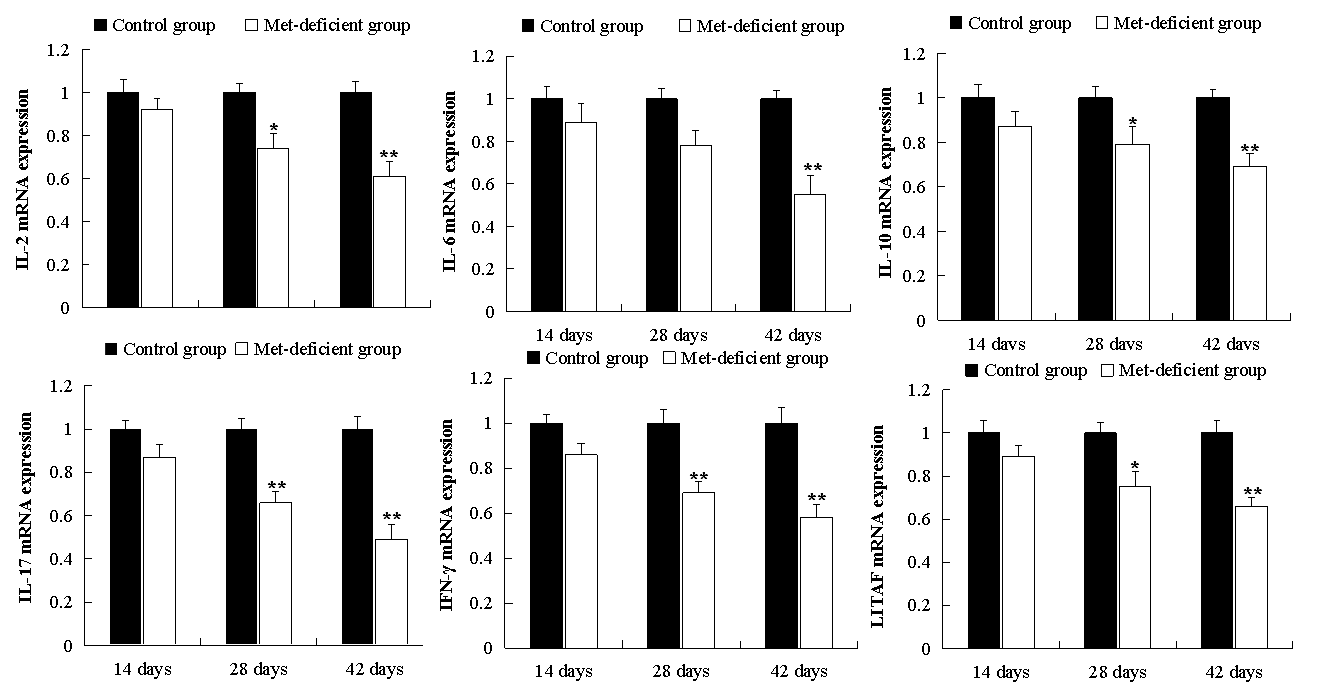
Suppl. Fig. S2. Changes of the IL-2, IL-6, IL-10, IL-17, IFN-γ, and LITAF MDNA expression in the ileum (n=6).**The similar expression trend of IL-2, IL-6, IL-10, IL-17, IFN-γ and LITAF MDNA were observed. Compared with the control group, the expression of IL-2, IL-6, IL-10, IL-17, IFN-γ and LITAF decreased or significantly decreased at the age of 28 and 42 days. (p < 0.05 or p < 0.01).
